# Validity of the Age-Adjusted Charlson Comorbidity Index on Clinical Outcomes for Patients with Nasopharyngeal Cancer Post Radiation Treatment: A 5-Year Nationwide Cohort Study

**DOI:** 10.1371/journal.pone.0117323

**Published:** 2015-01-24

**Authors:** Ching-Chieh Yang, Po-Chun Chen, Chia-Wen Hsu, Shih-Lun Chang, Ching-Chih Lee

**Affiliations:** 1 Department of Radiation Oncology, Chi-Mei Medical Center, Tainan, Taiwan; 2 Institute of Biomedical Sciences, National Sun Yat-Sen University, Kaohsiung, Taiwan; 3 Department of Radiation Oncology, Pingtung Christian Hospital, Pingtung, Taiwan; 4 Department of Medical Research, Dalin Tzu Chi Hospital, Buddhist Tzu Chi Medical Foundation, Chiayi, Taiwan; 5 Department of Otolaryngology, Chi-Mei Medical Center, Tainan, Taiwan; 6 Department of Otolaryngology, Dalin Tzu Chi Hospital, Buddhist Tzu Chi Medical Foundation, Chiayi, Taiwan; 7 Center for Clinical Epidemiology and Biostatistics, Dalin Tzu Chi Hospital, Buddhist Tzu Chi Medical Foundation, Chiayi, Taiwan; 8 Cancer Center, Dalin Tzu Chi Hospital, Buddhist Tzu Chi Medical Foundation, Chiayi, Taiwan; 9 School of Medicine, Tzu Chi University, Hualian, Taiwan; Taipei Medical University, TAIWAN

## Abstract

**Purpose:**

To characterize the impact of comorbidity on survival outcomes for patients with nasopharyngeal carcinoma (NPC) post radiotherapy (RT).

**Methods:**

A total of 4095 patients with NPC treated by RT or RT plus chemotherapy (CT) in the period from 2007 to 2011 were included through Taiwan’s National Health Insurance Research Database. Information on comorbidity present prior to the NPC diagnosis was obtained and adapted to the Charlson Comorbidity Index (CCI), Age-Adjusted Charlson Comorbidity Index (ACCI) and a revised head and neck comorbidity index (HN-CCI). The prevalence of comorbidity and the influence on survival were calculated and analyzed.

**Results:**

Most of the patients (75%) were male (age 51±13 years) and 2470 of them (60%) had at least one comorbid condition. The most common comorbid condition was diabetes mellitus. According to these three different comorbidity index (CCI, ACCI and HN-CCI), higher scores were associated with worse overall survival (P< 0.001). The Receiver Operating Characteristic (ROC) curve was used to assess the discriminating ability of CCI, AACI and HN-CCI scores and it demonstrated the predictive ability for mortality with the ACCI (0.693, 95% CI 0.670–0.715) was superior to that of the CCI (0.619, 95% CI 0.593–0.644) and HN-CCI (0.545, 95%CI 0.519–0.570).

**Conclusion:**

Comorbidities greatly inﬂuenced the clinical presentations, therapeutic interventions, and outcomes of patients with NPC post RT. Higher comorbidity index scores accurately was associated with worse survival. The ACCI seems to be a more appropriate prognostic indicator and should be considered in further clinical studies.

## Introduction

Nasopharyngeal carcinoma (NPC) is rare worldwide, but occurs with high prevalence in Chinese populations [1]. Taiwan has a high incidence of NPC: the annual incidence rate is 6.17 per 100,000 as compared with < 1 per 100,000 in Western countries [2]. Radiotherapy (RT) or concurrent with chemotherapy (CT) is the principle treatment modality and is associated with high cure rate. However, patients with NPC often have other comorbid diseases, which have a substantial and direct impact on treatment selection and subsequently outcomes [3,4].

Several instruments have been developed to assess, quantity and grade the degree of comorbid burdens using ordinal scales. One of the most widely applied is the Charlson Comorbidity Index (CCI), which has been extensively used to evaluate the impact of comorbidity in a variety of cancers and non-cancer conditions. The CCI was developed in 1987 and is a prognostic taxonomy that was initially developed to account for the inﬂuence of a patients’ adverse medical conditions in longitudinal studies [5–7] and has been validated in many clinical settings [8–10].This index is calculated by the summation of weight scores for 19 medical conditions and high scores were found to be associated with poorer prognosis. Due to age have been determined to be associated with overall survival, the CCI was modified by Charlson et al. in 1994 [11]. This modification called Age-Adjusted Charlson Comorbidity index (AACI) included the age of the patient as a correction variable of the final score of the Charlson index. Recently, a revised Charlson Comorbidity index used for head and neck cancer patients (HN-CCI) was reported by Bøjeet al [12]. In this large retrospective population based cohort study of 9388 HNSCC patients; the HN-CCI was able to stratify patients into prognostic groups based on six comorbidity conditions (congestive heart failure, cerebrovascular disease, chronic pulmonary disease, ulcer disease, liver disease, and diabetes). However, it is unknown whether the inﬂuence of comorbidities on clinical outcomes over patients with NPC due to the HN-CCI applies to NPC patients.

Thus, the present study, which was based on nationwide databases, focused on estimating the incidence of comorbidity and its impact on management in a cohort of patients with NPC consecutively planned and treated within RT or RT plus CT in Taiwan. A secondary objective was to compare these three different indices via the CCI, AACI and HN-CCI for predicting the outcomes in Asian NPC patients

## Materials and Methods

### Data source

This study used the Taiwan National Health Institute Research database (NHIRD), which was released by the Taiwan National Health Research Institute (NHRI) and available to all researchers in Taiwan. Taiwan initiated its National Health Insurance (NHI) program in March 1995. This system enrolls up to 99% of the Taiwanese population and contracts with 97% of all medical providers [13,14]. The database contains comprehensive information on all insured individuals, including sex, date of birth, residential or work area, dates of clinical visits, the International Classification of Diseases (Ninth Revision) Clinical Modification (ICD-9-CM) diagnostic codes, details of prescribed medications, expenditure amounts, and outcome at hospital discharge (ie, recovered, died, or transferred out). A random sample comprised of 1,000,000 people based on the 2005 reimbursement data was established for public access; the group did not significantly differ statistically from the larger cohort in age, gender, or health care costs, according to the Taiwan National Health Research Institute. The database contained a registry of contracted medical facilities, a registry of board-certified physicians, and monthly claims summary for all inpatient claims. Because these were de-identified secondary data, this study was exempt from full review by the internal review board.

### Study population

**Fig 1:**
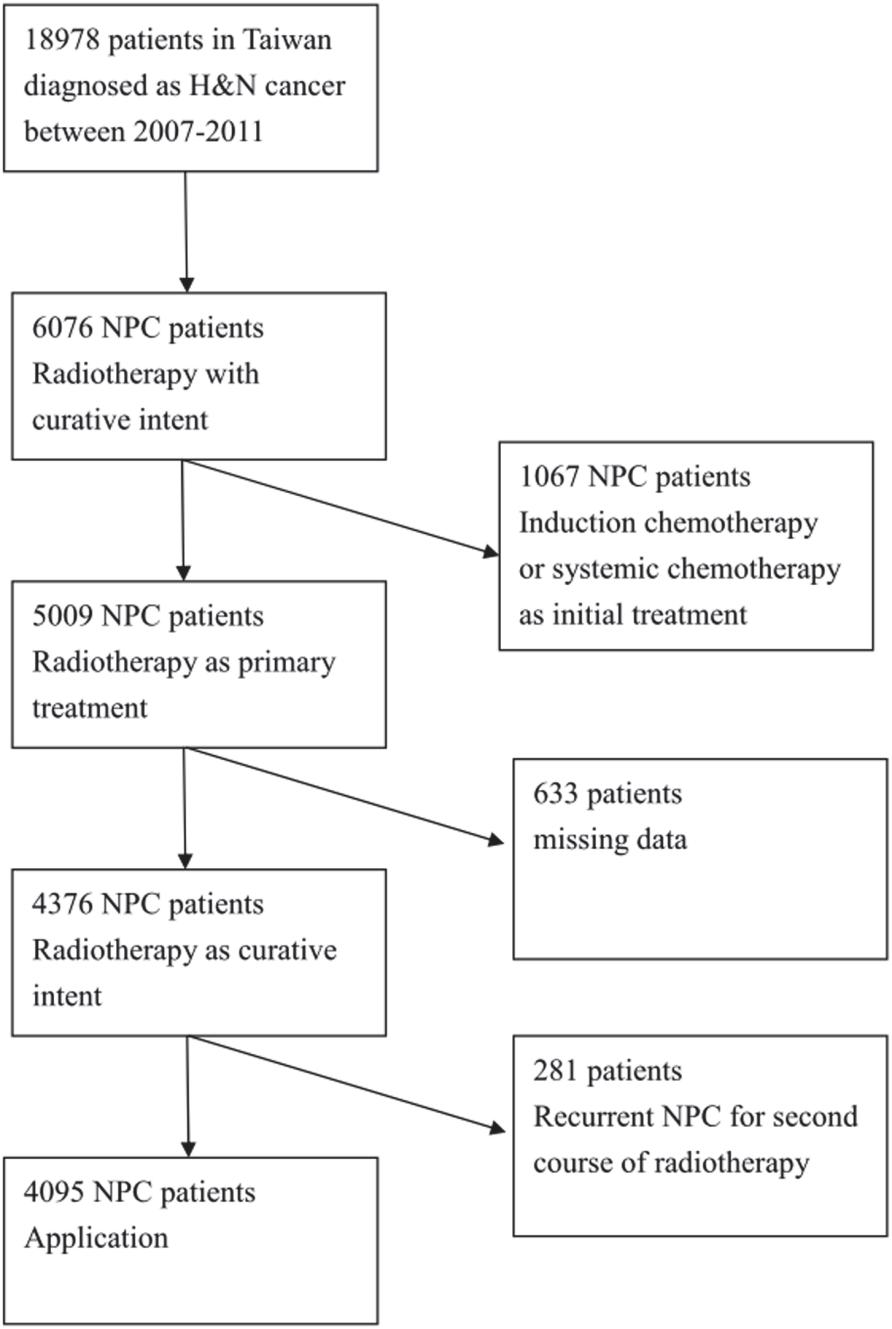
Patient selection flowchart.

All patients with NPC (International Classification of Disease, Ninth Revision, Clinical Modification codes147.0–147.9) who received curative RT or RT plus CT as initial treatment between the years 2007 and 2011 were identified. Initially, we inspected 6076 NPC patients who were treated with radiotherapy from Taiwan’s NHI research database. Taiwan’s NHI program covered 99% of the population after 2003 which charts reviews and patients interviews used to verify the accuracy of diagnosis and treatment coding. We excluded patients who were treated for second course of radiotherapy and who received systemic or induction chemotherapy as the initial treatment except those who received chemotherapy within 14 days prior to radiotherapy. Finally, after excluding the patients with missing data, a total of 4095 patients during this period were included in this study (Fig. 1). These NPC patients were then linked to the death data extracted from the records covering the years 2007 to 2013.

### Comorbidity assessment and analysis

Information on pre-existing comorbidity present before to the NPC diagnosis half a year was obtained and we utilized the comorbidity index developed by Charlson et al. to quantify baseline comorbidities available from the trial database [5,11]. This index is a weighted measure that incorporates 19 different medical categories and each weighted according to its potential to impact on mortality. Those with a relative risk below 1.5 were assigned a weight of 1; conditions with a risk of 1.5 to <2.5 a weight of 2; conditions with a risk of 2.5 to <3.5 a weight of 3; and metastatic tumors and AIDS were assigned a weight of 6. The final score was calculated for each patient by taking into account all comorbid conditions present when the index was applied. ACCI scores were calculated with additional points added for age. The age score involved including 1 point for each decade over the age of 40. The HN-CCI, another comorbidity index, is simple and has been validated for use in head and neck cancer [12]. To classify the patients in comorbidity groups, each of the identified six conditions was assigned one point each and final score was calculated, respectively.

### Statistical analysis

Patients were regularly followed up and assessed after RT until death or their latest follow-up appointment. Patients alive on the last day of follow-up were censored. The prevalence of the different comorbid conditions was calculated and described. Overall survival was estimated using the Kaplan—Meier method and the difference between groups were analyzed using log-rank test. The Cox proportional hazards regression model was used for multivariate analysis. Differences between variables with categorical data were examined using the chi-square test. A *P* value < 0.05 was regardedas statistically significant. Receiver Operating Characteristic (ROC) curves was generated to assess the accuracy and the predictive ability of each index for survival. It plots the false rate against the sensitivity to determine its overall accuracy in assessing the outcome. The area under the ROC curves for each index was compared with 95% confidence interval (CI). All data were analyzed using Statistical Package for Social Sciences (SPSS) version 15.

## Results

From 2007 to 2011, a total of 4095 patients diagnosed NPC receiving RT or RT plus CT were included in this analysis. These patients comprised 3080 men and 1015 women with a mean age of 51±13 years. Most patients (3077, 75.1%) received a combination of RT with systemic chemotherapy. The characteristics of this study population are summarized in Table1. The distribution of comorbidity for these patients based on the index is shown in Table 2. A total of 2470 (60.3%) had some form of non-cancer-associated comorbidities, with diabetes mellitus (10.4%), peptic ulcer disease (8.0%), chronic obstructive pulmonary disease (6.9%), cerebrovascular disease (5.5%), and congestive heart failure (4.5%) being the most common comorbid illnesses either alone or in combination. Other less common conditions included mild liver disease (3.8%), chronic kidney disease (2.3%), and peripheral vascular disease (1.3%).

**Table 1:** Patient Characteristics.

	Number of patients	%
Patients’ characteristics		
Age, mean(±SD)		51±13
Gender, male (%)	3080	(75.2)
Treatment		
RT^‡^ alone	1018	(24.9)
RT^‡^+ CT^+^	3077	(75.1)
CCI		
0	1625	(39.7)
1–5	802	(19.6)
More than 6	1668	(40.7)
ACCI		
0	1661	(40.6)
2–3	1357	(33.1)
More than 4	1077	(26.3)
HN-CCI		
0	3057	(74.7)
More than 1	1038	(25.3)
Socioeconomic status		
Low SES	1306	(31.9)
Moderate SES	1258	(30.7)
High SES	1531	(37.4)
Geographic Region		
Northern (*n* = 1745)	2071	(50.6)
Central (*n* = 502)	632	(15.4)
Southern (*n* = 946)	1259	(30.7)
Eastern (*n* = 95)	133	(3.2)

Abbreviations: nasopharyngeal carcinoma, NPC;+CT, chemotherapy; ‡RT, Radiation therapy; SD, standard deviation; CCI, Charlson Comorbidity Index; ACCI, Age-Adjusted Charlson Comorbidity Index; HN-CCI, revised head and neck Charlson Comorbidity Index

Demographic characteristics for NPC patients from 2007 to 2011 (n = 4095).

**Table 2:** Comorbidity Distribution Based on the Charlson Comorbidity Index (n = 4095).

Comorbidities	Charlson Comorbidity Index weight	Number of patients (%)
Myocardial infarction	1	25(0.6)
Congestive heart failure	1	183(4.5)
Peripheral vascular disease	1	54(1.3)
Cerebrovascular disease	1	226(5.5)
Dementia	1	11(0.3)
Chronic obstructive pulmonary disease	1	282(6.9)
Connective tissue disease	1	36(0.9)
Peptic ulcer disease	1	326(8.0)
Mild liver disease	1	154(3.8)
Diabetes mellitus without end‐organ damage	1	428(10.4)
Hemiplegia	2	18(0.4)
Moderate to severe chronic kidney disease	2	96(2.3)
Diabetes with end‐organ damage	2	20(0.5)
Solid tumor	2	462(11.3)
Leukemia	2	6(0.1)
Lymphoma	2	27(0.7)
Moderate to severe liver disease	3	50(1.2)
Metastatic solid tumor	6	1644(40.1)
Acquired immunodeficiency syndrome	6	3(0.1)

**Fig 2:**
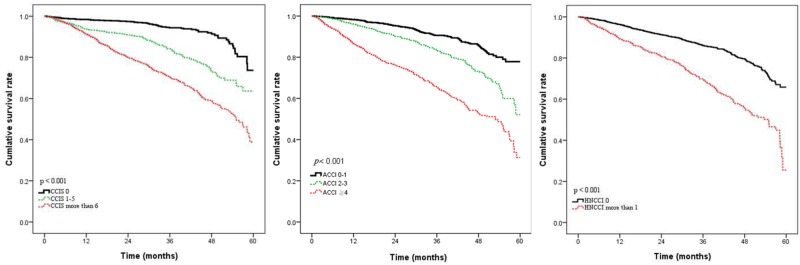
The influence of comorbidity according to CCI, ACCI and HN-CCI on patient survival.

A Charlson comorbidity score was calculated for each patient to classify comorbidity and grouped as having either no comorbidity (CCI = 0), moderate comorbidity (CCI = 1–5) or severe comorbidity (CCI ≥ 6). In total, no comorbidity was found in 39.7%, moderate comorbidity in 19.6% and severe comorbidity in 40.7% of these patients (Table 1). The inﬂuence of comorbidity on overall survival according to CCI is illustrated in Fig. 2. The presence of comorbidity was associated with increasing risk of overall death with 5-year OS of 77% (CCI = 0), 63% (CCI = 1–5), and 40% (CCI ≥6). Thus, the CCI was able to be used to classify patients in prognostic groups according to comorbidity. ACCI score was also calculated and these patients were dichtomized into three groups: 0–1, 2–3, and ≥4. In total, low score (0–1) was found in 40.6%, mild score (2–3) in 33.1% and severe score (≥4) in 26.3% of these patients (Table 1). The 5-year survival according to different degree of ACCI scores (low, mild, severe) was observed in Fig. 2 and showed comorbidity significantly correlated with poor outcome (*p* <0.001).

Based on the simple and main six medical conditions, the head and neck comorbidity index score (HN-CCI) were also calculated [12] by six conditions which was assigned one point each. In total, only 25.3% of the patients had one of these six comorbidities at time of diagnosis according to this revised index (Table 3). Applying the HN-CCI to these patients, the overall survival significantly decreased with increasing HN-CCI score (*P* < 0.001). The 5-year OS was 65% for HN-CCI = 0 and 24% for HN-CCI of more than 1 point. Thus, by using the HN-CCI to classify comorbidity, we were able to stratify patients to different prognostic groups and those patients with comorbidities had poor outcomes.

**Table 3:** Comorbidity Distribution Based on the HN-CCI (n = 4095).

Comorbidities	Charlson Comorbidity Index weight	Number of patients（％）
Congestive heart failure	1	54(1.3)
Cerebrovascular disease	1	167(4.1)
Chronic obstructive pulmonary disease	1	225(5.5)
Peptic ulcer disease	1	224(5.5)
Liver disease	1	230(5.6)
Diabetes	1	431(10.5)

**Fig 3:**
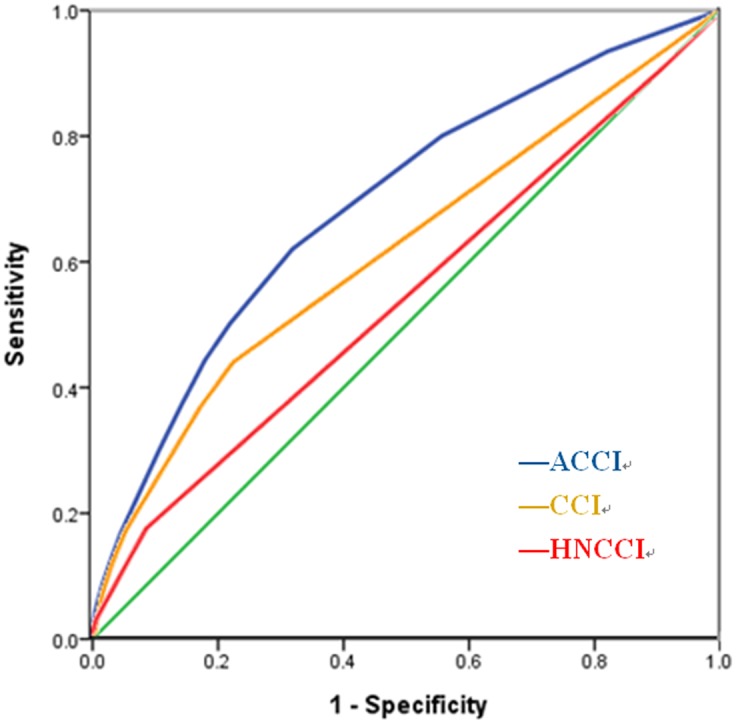
Receiver operating characteristic curve compared the discriminating ability for predicting survival of the ACCI (area = 0.693; 95% CI 0.670 to 0.715), CCI (area = 0.619; 95% CI 0.593 to 0.644) and HN-CCI (area = 0.545; 95% CI 0.519 to 0.570).

The ROC curve was used to assess the discriminating ability of CCI, ACCI and HN-CCI scores. The predictive ability for mortality of ACCI is superior to that of CCI and HN-CCI (Fig. 3). The areas under the ROC curves were 0.693 in ACCI (95% CI 0.670–0.715), 0.619 in CCI (95% CI 0.593–0.644) and 0.545 in HN-CCI (95% CI 0.519–0.570).

## Discussion

This population-based study demonstrated the importance of comorbidity in Asian NPC patients post radiotherapy. Our study on 4095 patients revealed that non-cancer-associated morbidity occurred in 2470 patients (60.3%) and was different from the comorbidity associated with smoking and alcohol abuse such as cardiovascular and hepatic diseases [15]. Based on different three comorbidity indexes, we found that worse overall survival was significantly associated with the degree of comorbidity.

Comorbidity can impact patient survival, which can lead to the development of non-cancer-associated competing mortality [16]. However, comorbidities may vary among people in different geographic regions or races. In patients who develop NPC that are not commonly alcohol, tobacco and betel quid chewing related, the prevalence of comorbidity is less than in patients with traditional risk factors for squamous cell carcinoma. In contrast to the typical head and neck subsites, few reports exist that have assessed the association of comorbidity with outcome in NPC patients.

Comorbidity indices are commonly used as synopsis for research and clinical purposes. The validity of a comorbidity index is based upon the assumption that increasing comorbidity will be associated with worse health outcomes. Assessment and measurement of comorbidity in head and neck squamous cell cancer patients have been actively investigated in the past and different methods/indices have been applied [17–20]. The prevalence of comorbid conditions in these studies ranged from 50% to 70% according to different comorbidity indices used. In this study, we used the Charlson Comorbidity index because it was the most extensively studied index [21], has been adapted for use with ICD-9 databases [22], and has predictive value for survival outcomes. This index was developed in 1984 by reviewing hospital charts for 559 hospital admissions in a New York Hospital. The association with 1-year all cause mortality was assessed and 19 comorbid conditions associated with mortality were found. Each condition was weighted and a comorbidity score could therefore be obtained. The index was validated in a cohort of 685 breast cancer patients. Since then, the index has been extensively used and was validated in a variety of cancer and non-cancer conditions, as well as in head and neck cancers [23]. One similar study was reported by Habbouset al. [24], who also used the Charlson Comorbidity index to evaluate comorbidity and prognosis in nasopharyngeal cancer patients. That study showed that patients with NPC were younger, more likely to be composed of never or fewer pack smokers and lighter drinkers (*P*<0.0001). Prevalence rates of comorbidity were 64%, 27%, 7%, and 2% for CCI of 0, 1, 2, and ≥3, respectively. After adjustment for either stage alone or stage and smoking and alcohol history, CCI was prognostic (HR, 2.93; 95% CI, 1.53–5.62; and HR, 2.82; 95% CI, 1.40–5.70, respectively; *P* = 0.001 for both models). The prevalence and severity rates of comorbidity observed in our patients were higher than the previous study. Most patients (60%) had comorbidities and most conditions were more severe (40% with CCI≥6). Cardiovascular and pulmonary diseases are the most common comorbidities occurring in Caucasians. However, the most frequent comorbid condition in the current study was diabetes mellitus, followed by peptic ulcer disease and pulmonary disease.

Some reports have proved the major role of comorbidity in the treatment outcomes of patients with NPC. A small study (n = 59) by Ramakrishnan et al. [25] reported no association of NPC with DSS. Another study found an association with OS and DSS, but the patient population was restricted to older patients [3]. Our data on 5-year survival rates, focusing on patients with NPC who were treated by RT or RT plus CT, showed those with moderate comorbidity (CCI 1–5) had 63%, severe comorbidity had a 40% (CCI ≥6) and those without comorbidities had 77%. These results demonstrate that comorbidity is an adverse survival prognostic factor. Because of the strong association between age and comorbidity, the impact on patient survival has been explored in many cancer types [6,26]. In this current study, we sought to evaluate the impact of comorbidity and age on survival and found that the CCI adjusted with age was an excellent assess tool than the original CCI.

The HN-CCI reported by Bøje et al. [12] has several advantages compared to the CCI for head and neck cancer patients. It is very simple to use and can be used both retrospectively from chart review or through health registries and prospectively in prospective trials. Furthermore, it has been developed in a population-based cohort of patients treated in a recent time-period and therefore it is considered valid at the present time. This index is specific to RT-treated HNSCC patients and should be a suitable and valid instrument to use for HNSCC patients treated with RT. However, in our results, the CCI or ACCI are better than HN-CCI for assessing comorbidity and for prognostic staging. This may due to the fact that NPC is often less related to tobacco, alcohol, and betal nut chewing and strongly associated with Epstein-Barr virus (EBV) [27]. Cigarette smoke and alcohol have long been associated with many other head and neck carcinomas, but their association with nasopharyngeal carcinoma has been controversial. Some studies have discussed that alcohol consumption and cigarette usage were not associated with nasopharyngeal carcinoma [28–30]. Nam et al. found that cigarette smoking and alcohol consumption are independent statistically significant risk factors for nasopharyngeal carcinoma in a case-control study by National Mortality Follow-Back Survey. These six comorbid conditions using in the HN-CCI were derived from head and neck squamous cell carcinoma patients caused by traditional risk factors and may be not suitable to evaluate NPC patients.

Our study has a few noteworthy limitations. First, we could not assess the relationship of comorbidity index to NPC stage because the staging information was not available from the database. Second, instead of cancer-specific survival rates, overall survival rate was used, because it was not possible to determine cause-specific mortality based on the registry data. A previous study by Bøje et al. also showed cancer specific death CCI was not influenced by comorbidity with 5-year disease specific survival. Third, the diagnosis of NPC and record of comorbid conditions are dependent factors on ICD codes. Different coding quality among each level of hospitals could result in bias. However, the NHI program in Taiwan reviewed charts to verify the accuracy of diagnosis and treatment coding. Given the robustness of the evidence and statistical analysis in this study, these limitations were unlikely to compromise our results.

## Conclusion

Our data demonstrate the significant survival impact of age and comorbidity on Asian patients with cancer of nasopharynx treated by RT or RT plus CT. The assessment of comorbidity is of great importance, and should optimize to treat patient’s health before RT. The CCI, ACCI and HN-CCI correlate well with survival and could be used for therapeutic decision-making. However, our analysis suggests the ACCI may be more useful and meaningful for further clinical studies.
